# Mastocytosis, MCAS, and Related Disorders—Diagnosis, Classification, and Therapy

**DOI:** 10.3390/ijms22095024

**Published:** 2021-05-10

**Authors:** Marek Niedoszytko, Peter Valent, Bogusław Nedoszytko

**Affiliations:** 1 Department of Allergology, Medical University of Gdansk, 80-211 Gdańsk, Poland; mnied@gumed.edu.pl; 2 Department of Internal Medicine I, Division of Haematology and Hemostaseology and Ludwig Boltzmann Institute for Hematology and Oncology, Medical University of Vienna, 18-20 Vienna, Austria; peter.valent@meduniwien.ac.at; 3 Department of Dermatology, Venerology and Allergology Medical University of Gdansk, 80-211 Gdańsk, Poland; 4 Invicta Fertility and Reproductive Center, Molecular Laboratory, Polna 64, 81-740 Sopot, Poland

Mastocytosis is a heterogeneous group of hematologic neoplasms defined by an accumulation of neoplastic mast cells (MC) in the skin, bone marrow, and other visceral organs. In many patients, signs and symptoms of MC activation are found. In particular, patients with systemic mastocytosis (SM) may variably suffer from cutaneous lesions, hypersensitivity (allergic) reactions, anaphylaxis, osteopenia or osteoporosis, gastrointestinal symptoms, cardiovascular symptoms, and psychological and neurological complaints. In advanced SM, patients also suffer from an impairment of organ function or even organ damage. The complex biology and pathology and the heterogeneous clinical courses require effective interdisciplinary collaborations and approaches and knowledge about diagnostic and therapeutic facilities in each case. Some of the patients are diagnosed with both, mastocytosis (SM) and MC activation syndrome (MCAS). The recent discovery of hereditary alpha tryptasemia (HαT) and epigenetic markers, and the development of new more effective KIT inhibitors have strengthened the diagnostic and therapeutic armamentarium in SM and MCAS [1,2,3].

This issue of IJMS summarises the current state of our knowledge on pathogenesis, genetic basis, clinical symptoms, laboratory assays, diagnostic criteria, classification, and diagnostic algorithms in mastocytosis and MCAS.

Three articles in this issue focus on the genetic basis of mastocytosis. One article presents a genome-wide association study (GWAS) investigating associations between mastocytosis and SNPs. The results of this study suggest that there is an association between mastocytosis and 9 SNPs which were not described in mastocytosis contexts so far. Four SNPs were more prevalent (in *ABCA2*, *OTX2-AS1, HLA-V,* and *PDE4DIP* genes) and 5 were less prevalent (in *RPTN, CYP2B6, OR51Q1, FTCD,* and rs9828758 near *RP11* genes) in mastocytosis patients compared to a control cohort. The genetic regulation of tryptase production and symptoms of HαT and implications in mastocytosis are also discussed in this article. HαT is a hot topic in the fields of allergology, dermatology, gastroenterology, rheumatology, and haematology. About 4–6% of the general population carry germline *TPSAB1*-α copy number gains (2α:3β, 3α:2β or more α-extra-copies), resulting in elevated basal serum tryptase levels. Although many carriers of HαT appear to be asymptomatic, a number of more or less specific symptoms have been associated with HαT. Recent studies revealed a significantly higher HαT prevalence (15–20%) in patients with SM and an association with concomitant severe Hymenoptera venom-induced anaphylaxis. Moreover, HαT seems to be more common in patients with idiopathic anaphylaxis and MCAS patients than in controls. 

Additional articles reviewed diagnostic challenges in mastocytosis and MCAS, specific issues in paediatric patients, response criteria in advanced mastocytosis, and personalised approaches to the treatment. One article described current methods used in hypersensitivity and allergy in MCAS and mastocytosis. Specific topics in *Hymenoptera* venom anaphylaxis, drug, food, and inhalant reactions were also discussed with an emphasis on novel diagnostic tests including microarrays, recombinant allergen analysis, basophil activation tests, HαT diagnosis. Specific issues in paediatrics were discussed, including the symptoms of MC mediator release and anaphylaxis in children with mastocytosis and MCAS with emphasis on risk factors, triggers, and management, and current pathogenic concepts, including genetic alterations. Skin lesions, algorithm of diagnosis in children with mastocytosis, treatment and prognosis were also reviewed and discussed.

Finally the crucial aspects of advanced mastocytosis and of treatment response criteria in these patients were addressed in a paper by Shomali and Gotlib. They discussed the current status treatment of advanced SM with tyrosine kinase inhibitors (TKI), including midostaurin and avapritinib. In addition the use of novel TKI and other novel drugs in patients with advanced SM were discussed. 

We hope that the content of *IJMS* Special Issue “Mastocytosis, MCAS, and Related Disorders–Diagnosis, Classification and Therapy” will be attractive for readers, indicating new ideas and fields of research.
